# PDK1-dependent metabolic reprogramming regulates stemness and tumorigenicity of osteosarcoma stem cells through ATF3

**DOI:** 10.1038/s41419-025-07903-7

**Published:** 2025-07-29

**Authors:** Kazuya Tokumura, Kazuya Fukasawa, Jiro Ichikawa, Koki Sadamori, Manami Hiraiwa, Eiichi Hinoi

**Affiliations:** 1 Department of Bioactive Molecules, Pharmacology, Gifu Pharmaceutical University, Gifu, Japan; 2 Department of Orthopaedic Surgery, Interdisciplinary Graduate School of Medicine, University of Yamanashi, Chuo, Japan; 3 United Graduate School of Drug Discovery and Medical Information Sciences, Gifu University, Gifu, Japan; 4 Center for One Medicine Innovative Translational Research (COMIT), Division of Innovative Modality Development, Institute for Advanced Study, Gifu University, Gifu, Japan

**Keywords:** Bone cancer, Bone cancer

## Abstract

Osteosarcoma stem cells (OSCs) are characterized by their self-renewal and multilineage differentiation abilities, contributing to osteosarcoma malignancy. The Warburg effect describes cancer cells’ preference for glycolysis over mitochondrial oxidative phosphorylation (OXPHOS) for energy production. Unlike differentiated cancer cells, cancer stem cells exhibit unique and diverse metabolic properties depending on the context. This study investigated the metabolic reliance of OSCs and related genes through in silico analyses of clinical osteosarcoma specimens and in vitro and in vivo genetic and pharmacological analyses. Glycolysis and OXPHOS pathways were more active in OSCs than in non-OSCs at single-cell resolution. Pyruvate dehydrogenase kinase 1 (PDK1), a key enzyme balancing glycolysis and OXPHOS, was upregulated in OSCs and correlated with poor prognosis in patients with osteosarcoma. Genetic inhibition of *PDK1* via RNA interference reduced OSC stemness, glycolysis, and heterotopic tumor formation. Pharmacological inhibition of PDK1 mirrored these genetic effects and repressed orthotopic tumor burden and pulmonary metastasis. Activating transcription factor 3 (ATF3) was identified through screening as a downstream factor of PDK1-regulated OSC properties. *ATF3* overexpression reversed the stemness reduction caused by *PDK1* deficiency through, at least in part, activating the TGF-β/Smad pathway without affecting the metabolic balance. ATF3 expression, glycolysis, and stemness were significantly induced by wild-type *PDK1* overexpression but not by a kinase-dead *PDK1* mutant in OSCs. Pharmacological inhibition of glycolysis counteracted the upregulation of ATF3 expression and increased stemness in OSCs by *PDK1* overexpression. These findings indicate that PDK1 fine-tunes metabolic balance to govern OSC stemness and tumorigenicity through, at least in part, modulating ATF3/TGF-β/Smad pathway, suggesting a potential therapeutic approach for targeting OSCs in osteosarcoma.

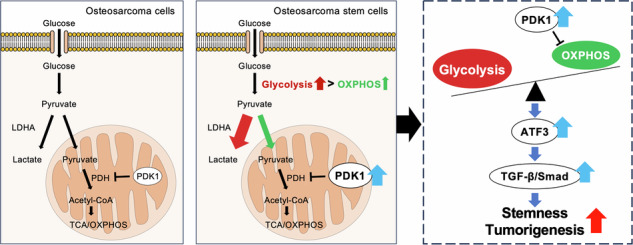

## Introduction

Osteosarcoma, the most common primary malignant bone tumor, poses a significant risk of bone and lung metastases [1–3]. Osteosarcoma incidence peaks in adolescents, young adults, and adults aged 65 and above [1–3]. It is highly malignant, invasive, progresses rapidly, and has a high mortality rate [1]. The 5-year survival rate is about 70% without metastases but drops to 25% with metastatic disease [2]. Chemotherapy with drugs like cisplatin, doxorubicin, and methotrexate improves survival but is not suitable for all patients [3]. The exact cell origin of osteosarcoma is unclear, but it likely arises from osteoblast lineage cells derived from mesenchymal stem cells (MSCs) [1]. Osteosarcoma stem cells (OSCs), defined by their self-renewal and multilineage differentiation abilities, significantly contribute to tumor initiation, recurrence, metastases, and chemoresistance, highlighting the need for novel OSC-targeted therapies [4–6].

Cancer cells produce energy differently from normal cells. The Warburg effect describes cancer cells’ preference for glycolysis over mitochondrial oxidative phosphorylation (OXPHOS) for energy, even with sufficient oxygen supply [7–9]. Unlike differentiated cancer cells, cancer stem cells (CSCs) exhibit unique and diverse metabolic properties based on cancer type, tissue type, and microenvironment [10]. OXPHOS is the primary energy source for many CSC types, including those in leukemia, glioblastoma, and lung, breast, ovarian, and pancreatic cancers [11–16]. However, some CSC types prefer glycolysis to maintain their stem cell traits [17–19]. Contradictory findings exist for the same tumor types (e.g., glioblastoma and breast cancer) regarding CSCs’ reliance on glycolysis or OXPHOS for sustaining their characteristics and tumorigenicity [12, 14, 17, 18, 20, 21].

During glycolysis, glucose is converted to pyruvate, which further undergoes anaerobic or aerobic conversion to lactate or acetyl-coenzyme A (acetyl-CoA) [22]. Pyruvate dehydrogenase kinase 1 (PDK1), a Ser/Thr kinase, serves as a gatekeeper in balance between glycolysis and OXPHOS [23, 24]. Phosphoglycerate kinase 1 (PGK1) phosphorylates PDK1 at Thr338, activating PDK1 to phosphorylate and inhibit the pyruvate dehydrogenase (PDH) complex, responsible for converting pyruvate to acetyl-CoA [25]. PDK1 is associated with cell proliferation, metastasis, and poor prognosis in breast cancer, glioblastoma, and leukemia, among others [26–28]. In addition, it regulates energy metabolism in normal stem cells such as hematopoietic stem cells (HSCs) and is enriched in breast CSCs, playing a key role in their reprogramming [29, 30]. However, the role of PDK1 in OSCs and its mechanism in maintaining stemness and tumorigenicity remain unclear.

Targeting OSCs is a promising strategy for improving osteosarcoma treatment [5]. Understanding the energy metabolic preferences and underlying molecular mechanisms of OSCs is essential for developing new osteosarcoma therapies. Although some studies have explored metabolic pathways in OSCs, data on their metabolic dependency at single-cell resolution of clinical specimens and the genes involved in osteosarcoma malignancy are limited [31, 32]. This study aimed to investigate the energy metabolism preferences and candidate genes in OSCs associated with poor clinical outcomes in osteosarcoma through bioinformatics analyses of clinical osteosarcoma specimens at single-cell resolution and in vitro and in vivo genetic and pharmacological analyses.

## Material and methods

### Cell culture

HEK293T cells (RIKEN Cell Bank, BioResource Center, Tsukuba, Japan) were cultured in DMEM (FUJIFILM Wako Pure Chemical Co., Osaka, Japan) with 10% fetal bovine serum (FBS; Hyclone, Logan, UT, USA) and 1% penicillin/streptomycin (Thermo Fisher Scientific, Waltham, MA, USA) at 37 °C in an atmosphere containing 5% CO_2_. The human osteosarcoma cell lines SJSA1, HOS, and 143B-luciferase cells (143B-luc), which fluoresce in the presence of the D-luciferin substrate, were kindly provided by Dr. Ichikawa (University of Yamanashi, Yamanashi, Japan) [33]. The other cell lines used in this study were 143B (ATCC, Manassas, VA, USA), MG-63 (RIKEN Cell Bank, Tsukuba, Japan), Saos-2 (Cell Resource Center for Biomedical Research, Institute of Development, Aging and Cancer, Tohoku University, Japan), and Hu-09 (JCRB Cell Bank, Osaka, Japan). These cells were maintained in DMEM supplemented with 10% FBS, 110 μg/mL sodium pyruvate (FUJIFILM Wako Pure Chemical), and 1% penicillin/streptomycin at 37 °C in a 5% CO_2_ atmosphere. For OSCs, cells were cultured in tumorsphere medium (DMEM/F12 (FUJIFILM Wako Pure Chemical) with B27 supplement without vitamin A (Thermo Fisher Scientific), GlutaMAX (Thermo Fisher Scientific), 20 ng/mL recombinant human EGF (FUJIFILM Wako Pure Chemical), 20 ng/mL recombinant human basic FGF (FUJIFILM Wako Pure Chemical), and 1% penicillin/streptomycin) at 37 °C in a 5% CO_2_ atmosphere [34].

### Lentiviral supernatant preparation and infection

HEK293T cells were transfected with lentiviral, packaging, and envelope plasmids using calcium phosphate to produce lentiviral particles [35]. Fourteen hours post-transfection, the medium was replaced with fresh DMEM containing 10% FBS. Forty-eight hours later, the lentiviral supernatant was collected, infecting 143B and MG-63 cells for 24 h. The following plasmids were used: short hairpin (sh) *PDK1*#1 (Sigma-Aldrich, St. Louis, MO, USA, TRCN0000006261), sh*PDK1*#2 (Sigma-Aldrich, TRCN0000006262), sh*Activating transcription factor 3* (*ATF3*)#1 (Sigma-Aldrich, TRCN0000013572), sh*ATF3*#2 (Sigma-Aldrich, TRCN0000329689), pLKO.1 puro plasmid (Addgene, #8453), control vector (Vector Builder, Chicago, IL, USA, # VB010000-9389rbj), *PDK1*^WT^ (Vector Builder, VB900004-5579neb), and *ATF3* (Vector Builder, #VB900000-5162ype). The *PDK1*^T358A^ mutant was generated from *PDK1*^WT^ using the QuikChange II XL site-directed mutagenesis kit (Agilent, Santa Clara, CA, USA). Nucleotide sequences of *PDK1*^WT^ and *PDK1*^T358A^ are analyzed using FinchTV software (ver. 1.5.0, Geospiza Inc, Seattle, WA, USA).

### Western blotting

Proteins were extracted by lysing cells in a buffer containing 1% nonidet P-40 and a protease inhibitor cocktail. Proteins were separated via SDS-PAGE, transferred onto PVDF membranes, and subjected to blotting [36]. The following antibodies were used. Primary antibodies: anti-PDK1 (1:1000, Cell Signaling Technology, CST, Danvers, MA, USA, #3820), anti-PDH (1:1000, CST, #2784), anti-p-PDH (1:1000, CST, #31866), anti-SOX2 (1:1000, CST, #14962), anti-c-Myc (1:1000, CST, #5605), anti-ATF3 (1:500, Santa Cruz Biotechnology, SCBT, TX, USA, #sc-188), anti-Smad3 (1:1000, CST, #9523), anti-p-Smad3 (1:1000, CST, #9520), anti-TGF-β1 (1:1000, Abcam, Fremont, CA, USA, #ab215715), and anti-β-actin (1:2000, SCBT, #sc-47778). Secondary antibodies: antirabbit IgG, HRP-linked (1:2000, CST, #7074), and antimouse IgG, HRP-linked (1:4000, CST, #7076). Protein levels were quantified using ImageJ.

### Sphere formation assay and in vitro limiting dilution assay

Cells were seeded at 1000 cells per well in ultralow attachment 96-well plates (Corning Incorporated, Corning, NY, USA) and cultured in tumorsphere medium with 1% methylcellulose (FUJIFILM Wako Pure Chemical) [34]. After 7 days, the number of spheres formed was counted. Sphere formation efficiency was assessed by counting spheres with >50 μm diameter. For in vitro limiting dilution assays, cells were plated in 96-well plates at densities of 1, 5, 10, 20, 40, or 80 cells/well (10 replicates each). The presence of a sphere was observed on day 7 and was analyzed using the “statmod” package (ver. 1.5.0) in R software (ver. 4.3.0).

### MTT, apoptosis, and wound healing assays

MTT, apoptosis, and wound healing assays were performed to assess cell characteristics [37]. For the MTT assay, 200 μL of 0.5 mg/mL MTT solution in PBS was added to each well on each measurement day and incubated for 4 h. Subsequently, 200 μL of 0.04 mol/L HCl in isopropanol was added to dissolve formazan crystals, and absorbance was measured at 550 nm with a microplate reader. For the wound healing assay, wounds were created using sterile 100 μL pipette tips, and images were taken at 0 and 24 h using a BZ-X810 Analyzer (KEYENCE, Osaka, Japan). The wound closure area-to-wound area ratio (migration rate) was calculated using ImageJ. For the apoptosis assay, cells were stained with PE-labeled Annexin V (1:50, BD Biosciences, BD, San Jose, CA, USA, #560930) and 7-AAD (1:1000, BD, #559925) for 30 min at 4 °C in the dark, followed by analysis using a CytoFLEX S flow cytometer (Beckman Coulter, Brea, CA, USA).

### Measurement of glucose uptake, lactate production, and the ADP/ATP ratio

Glucose uptake, lactic acid production, and the ADP/ATP ratio were assessed using Glucose Assay Kit-WST (Dojindo, Kumamoto, Japan), Lactate Colorimetric/Fluorometric Assay Kit (BioVision, SF, USA), ADP/ATP Ratio Assay Kit (Sigma-Aldrich), and Glycolysis/OXPHOS Assay Kit (Dojindo) following the manufacturers’ protocols.

### Chemicals and reagents

2,2-Dichloroacetophenone (DAP) was obtained from Tokyo Chemical Industry (Tokyo, Japan), and FX11 was obtained from Selleck (Houston, TX, USA).

### In vivo experiments

To generate heterotopic xenograft mouse model, 143B cells (5 × 106) were subcutaneously injected into 4-week-old female BALB/cSlc-nu/nu mice (SLC, Hamamatsu, Japan). Tumor volume (calculated as length × width^2^/2) was measured every 5 days with a digital caliper, with an endpoint set at a tumor diameter of 20 mm. For immunohistochemistry, the following antibodies were used: anti-SOX2 (1:300, CST, #14962) as a primary antibody, Alexa Fluor 546 goat antirabbit IgG (1:400, Invitrogen, CA, USA, #A11035) as a secondary antibody, and DAPI (1:1000, Dojindo, #340-07971) for nuclear staining. Immunofluorescence was visualized using a BZ-X800 Analyzer (KEYENCE). To generate orthotopic xenograft mouse model, 143B-luc cells (1 × 10⁴) were intratibially injected into 4-week-old female BALB/cSlc-nu/nu mice. To generate pulmonary metastasis mouse model, 143B-luc cells (1 × 10⁶) were intravenously injected into 4-week-old female BALB/cSlc-nu/nu mice. Bioluminescence imaging was performed on days 7 and 14 post inoculation using the IVIS Lumina II system (PerkinElmer, Waltham, MA, USA). D-luciferin (150 mg/kg, Wako) was intraperitoneally administered 10 min prior to imaging. The study protocol meets the guidelines of the Japanese Pharmacological Society and was approved by the Committee for the Ethical Use of Experimental Animals at Gifu Pharmaceutical University and Gifu University.

### scRNA-seq data analysis

The GSE152048 and GSE179681 datasets was analyzed using the “Seurat” package (ver. 5.1.0) in R software (ver. 4.3.0) [38, 39]. For analysis of the GSE152048 dataset, seven samples were used: five patients with conventional/primary types (BC2, BC3, BC5, BC6, BC16), one patient with conventional/metastatic types (BC10), and one patient with conventional/recurrent types (BC11). During preprocessing, cells with mitochondrial RNA content ≥10%, expressed genes <300 were filtered as low-quality. Potential doublets were removed using the “DoubletFinder” package (ver. 2.0.4). Normalization was performed using SCTransform function. To remove the batch effects, integration of the 7 sample datasets was performed using IntegrateLayers function. For analysis of the GSE179681 dataset, 8 samples were used: 4 samples with primary types (143B, NCH-OS-2, NCH-OS-7, OS17), and 4 samples with metastatic types (143B, NCH-OS-2, NCH-OS-7, OS17). During preprocessing, cells with mitochondrial RNA content ≥15%, expressed genes <300 were filtered as low-quality. Potential doublets were removed using the “DoubletFinder” package (ver. 2.0.4). Normalization was performed using SCTransform function. To remove the batch effects, integration of the 8 sample datasets was performed using IntegrateLayers function. single-sample Gene Set Enrichment Analysis (ssGSEA) was performed on tumor cell clusters using the “GSVA” package (ver. 1.50.5) [40, 41]. For each of the primary, metastasis, and recurrent types, OSC cells were identified as the top 1% in ssGSEA scores, utilizing the MALTA_CURATED_STEMNESS_MARKERS gene set. Differentially expressed genes (DEGs; *p* < 0.05) were identified between groups with the Wilcoxon rank-sum test using the “presto” package (ver 1.0.0). Gene set enrichment analysis (GSEA) was performed with the “clusterProfiler” package (ver 4.8.3), with gene sets downloaded through the “msigdbr” package (ver 7.5.1) [42, 43]. Results were visualized using the “enrichplot” package (ver 1.20.3) and “ggplot2” (ver 3.4.4).

### Bulk RNA-seq data analysis

For 143B OSCs RNA-seq (GSE297211), cells were lysed and total RNA was extracted using FastGene RNA Premium Kit (NIPPON Genetics, Tokyo, Japan) per the manufacturer’s instructions. Paired-end sequencing was performed on the Illumina NovaSeq 6000 platform.

For the RNA-seq analysis of GSE297211, PRJNA539828, and GSE126209 datasets, trimming was conducted using “Trimmomatic” (ver. 0.39), followed by quality checks with “FASTQC” (ver 0.12.1) [44]. Alignment to the Homo sapiens genome (GRCh38.p14) was performed using “STAR” (ver. 2.7.11a), and expression levels were calculated from the resulting BAM files using “RSEM” (ver. 1.3.3).

### Survival analysis

Survival data for osteosarcoma patients were obtained from the TARGET-OS, TCGA-SARC, and GSE21257 datasets [45]. Survival analysis was carried out using the log-rank test in the “survival” package (ver. 3.5-5), and Kaplan–Meier curves were generated using the “survminer” package (ver. 0.4.9).

### Statistical analysis

Statistical analyses were performed using R software (ver. 4.3.0), and significance was indicated in the results (*p < 0.05, **p < 0.01, ***p < 0.001, ^#^*p* < 0.05, ^##^*p* < 0.01, ^###^*p* < 0.001). Unless otherwise specified, results are presented as mean ± standard deviation (SD). Comparisons between two groups were evaluated using Student’s *t*- or Wilcoxon test, whereas comparisons among three or more groups were conducted using one-way or two-way ANOVA followed by Tukey–Kramer test, or one-way ANOVA followed by Dunnett’s test. A *p*-value < 0.05 was considered statistically significant.

## Results

### Enrichment of glycolysis and OXPHOS pathways in OSC

**Fig. 1:**
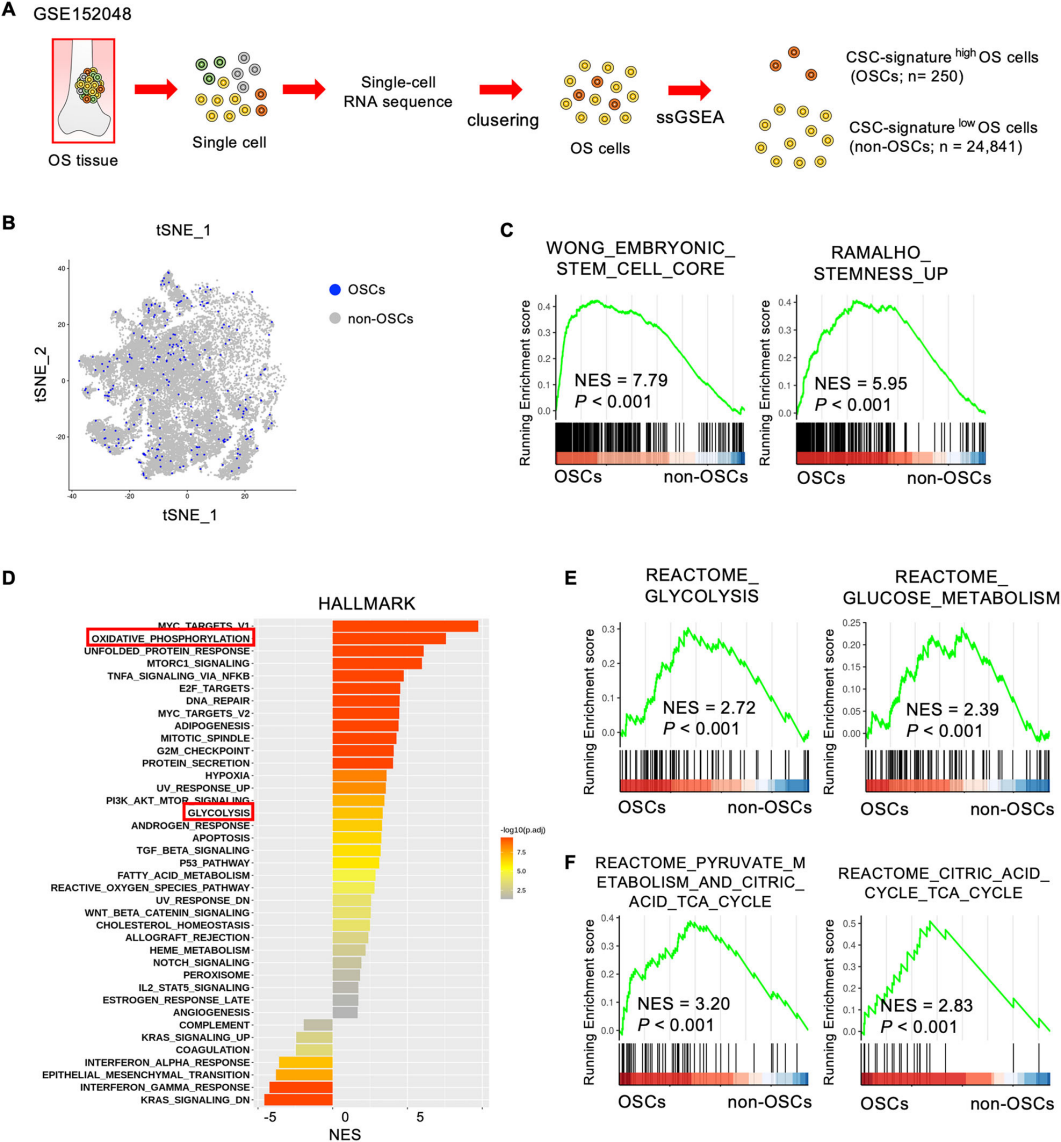
Energy metabolic properties of OSCs analyzed via scRNA-seq. **A** Schematic illustrating scRNA-seq analysis performed using the GSE152048 dataset; **B** t-SNE plot distinguishing OSCs (*n* = 250) and non-OSCs (*n* = 24,841) classified by ssGSEA; **C** GSEA plot confirming the enrichment of stemness-related gene sets in OSCs; **D** GSEA results for HALLMARK gene sets identify pathways enriched in OSCs; GSEA of REACTOME gene sets reveal significant enrichment of glycolysis (**E**) and oxidative phosphorylation (OXPHOS) (**F**) pathways in OSCs.

We analyzed a scRNA-seq dataset (GSE152048) of clinical osteosarcoma specimens from five conventional and primary patients to profile the energy metabolic properties of OSCs (Fig. 1A) [38]. Six clusters were identified through t-SNE analysis based on genetic profiles (Supplementary Fig. 1A, B). Canonical markers were used to annotate different cell types: malignant cells (*COL1A1*, *IBSP*), myeloid cells (*CD74*, *CD14*), osteoclasts (*CTSK*, *MMP9*), pericytes (*RGS5*, *ACTA2*), endothelial cells (*PECAM1*, *VWF*), and tumor-infiltrating lymphocytes (*CD3D*, *NKG7*) (Supplementary Fig. 1C). The osteosarcoma cell population was divided into OSCs and non-OSCs by ssGSEA (Fig. 1B and Supplementary Fig. 1D). The enrichment of two gene sets associated with “stemness” in OSCs was confirmed by GSEA (Fig. 1C), defining these cells as the OSC population. We performed GSEA on HALLMARK gene sets to characterize OSCs, finding enrichment in “glycolysis” and “OXPHOS” gene sets in OSCs compared to non-OSCs (Fig. 1D). Similarly, GSEA in the REACTOME gene sets showed that both “glycolysis” and “OXPHOS” were enriched in these pathways in OSCs (Fig. 1E, F). Thus, scRNA-seq analysis of clinical osteosarcoma specimens suggests activation of glycolysis and OXPHOS pathways in OSCs.

### *PDK1* is upregulated in OSC and linked to prognosis in patients with osteosarcoma

**Fig. 2:**
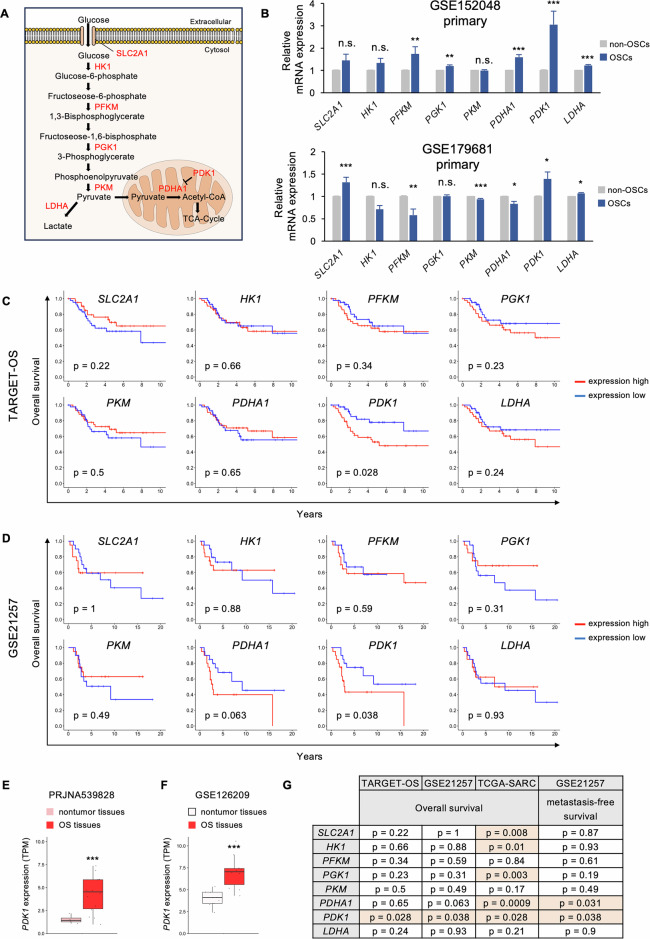
Upregulation of *PDK1* expression in OSCs and its correlation with poor prognosis in patients with osteosarcoma. **A** Schematic representing genes involved in early energy metabolism stages; **B** mRNA expression levels of genes related to early energy metabolism pathways in OSCs (*n* = 250) and non-OSCs (*n* = 24,841) from the GSE152048 dataset, and in OSCs (*n* = 167) and non-OSCs (*n* = 16,544) from the GSE179681 dataset (Wilcoxon test, mean ± standard error); **C**, **D** Kaplan–Meier survival curves showing overall survival for patients with osteosarcoma with high and low expression of early energy metabolism genes; **C** TARGET-OS cohort (high; *n* = 43, low; *n* = 42, Log-rank test); **D** GSE21257 cohort (high; *n* = 20, low; *n* = 20, Log-rank test). **E**, **F** *PDK1* mRNA expression levels in osteosarcoma and nontumor tissues; **E** PRJNA539828 dataset (osteosarcoma tissues; *n* = 16, nontumor tissues; *n* = 4, Wilcoxon test); **F** GSE126209 dataset (osteosarcoma tissues; *n* = 10, nontumor tissues; *n* = 9, Wilcoxon test); **G** summary table illustrating the relationship between early energy metabolism gene expression and survival outcomes; *p < 0.05, **p < 0.01, ***p < 0.001.

Using scRNA-seq data (GSE152048) and (GSE179681), we identified DEGs in early energy metabolic pathways in OSCs (Fig. 2A). In OSCs of primary samples, the expression levels of *PFKM, PGK1*, *PDHA1*, *PDK1*, and *LDHA* were significantly upregulated according to GSE152048, whereas the expression levels of *SLC2A1, PDK1*, and *LDHA* were increased; however, the expression levels of *PFKM*, *PKM*, and *PDHA1* were decreased according to GSE179681 (Fig. 2B). Contrary to the primary samples, the expression level of *PFKM* was increased in OSCs of metastatic samples, and the expression levels of *HK1* and *PFKM* were increased in OSCs of recurrent samples according to GSE152048 (Supplementary Fig. 2A). The expression levels of *PDHA1* and *LDHA* were significantly decreased in OSCs of metastatic samples according to GSE179681 (Supplementary Fig. 2B). The association of the expression levels of these metabolic genes with the survival Kaplan–Meier analysis of patients was assessed using the TARGET-OS database. It showed that higher *PDK1* expression correlated with shorter survival than those with lower *PDK1* expression being the only gene correlating significantly with the prognosis of patients with osteosarcoma (Fig. 2C). High *PDK1* expression also predicted poorer overall and metastasis-free survival in patients with osteosarcoma according to the microarray dataset (GSE21257) (Fig. 2D and Supplementary Fig. 2C) and was linked to poor prognosis of patients with soft tissue sarcoma (TCGA-SARC) (Supplementary Fig. 2D). *PDK1* was significantly upregulated in clinical osteosarcoma tissues compared to nontumor tissues in two bulk RNA-seq datasets (PRJNA539828, GSE126209) (Fig. 2E, F). Among the early stage of the energy metabolic pathway genes, *PDK1* was the only gene that fulfilled the significantly upregulated in OSCs and the consistently correlated with poor prognosis in patients with osteosarcoma across cohorts (Fig. 2G), suggesting its potential role in osteosarcoma progression.

### OSC favors glycolysis via PDK1 overexpression

**Fig. 3:**
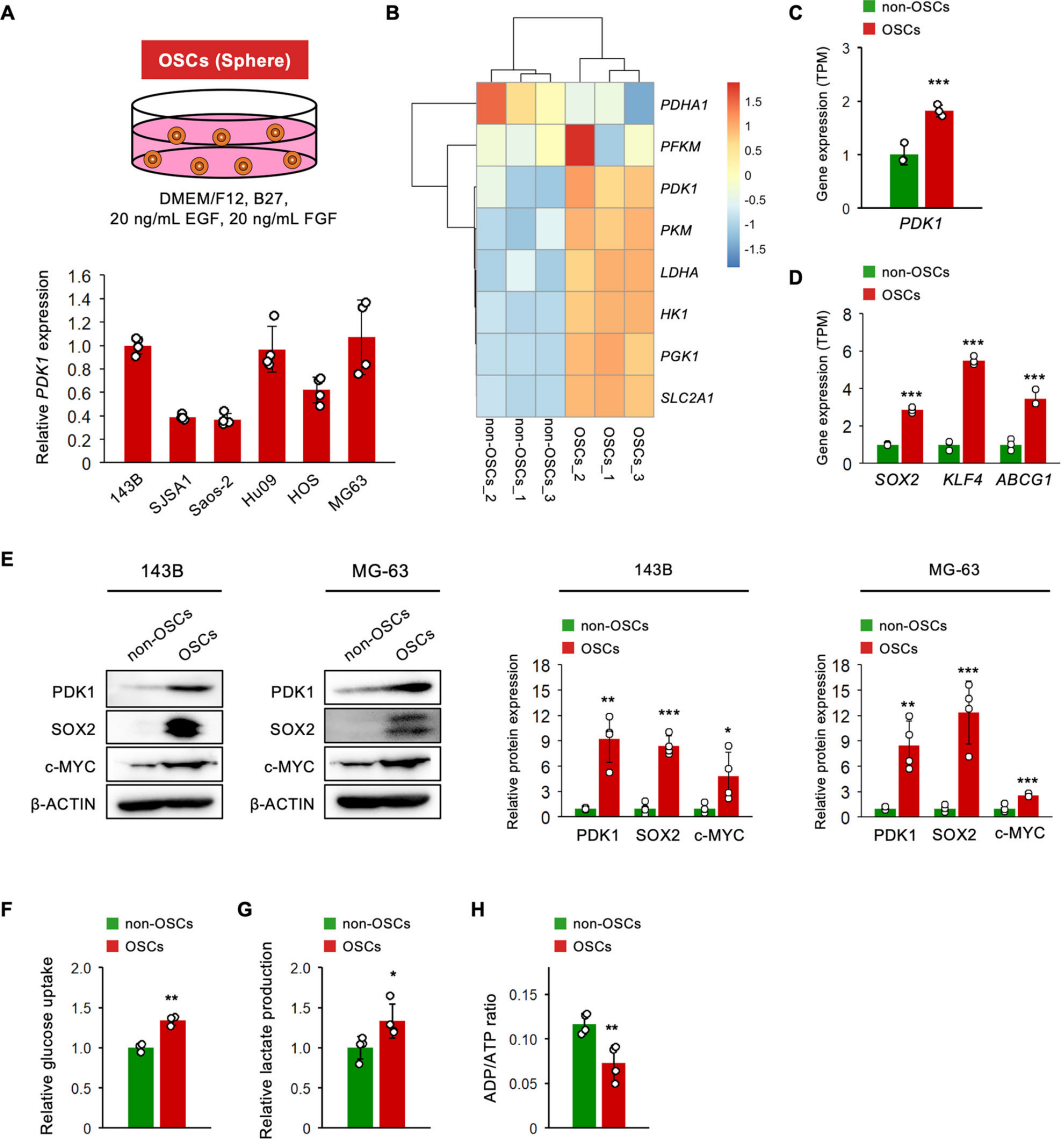
Preference of OSCs for glycolysis through PDK1 upregulation. **A** Schematic of culture condition for OSCs and mRNA expression level of *PDK1* in OSCs of different osteosarcoma cell lines (*n* = 4); **B**–**D** Bulk RNA-seq analysis comparing OSCs (*n* = 3) and non-OSCs (*n* = 3) derived from 143B cells; **B** heatmap illustrating expression levels of genes linked to early energy metabolism; **C** mRNA expression levels of *PDK1* (Student’s *t*-test); **D** mRNA levels of stem cell marker genes (Student’s *t*-test); **E** western blot analysis of PDK1 and stem cell markers in OSCs and non-OSCs derived from 143B and MG-63 cells (*n* = 4, Student’s *t*-test); **F**–**H** analysis of glycolytic activity in OSCs and non-OSCs from 143B cells; **F** glucose uptake (*n* = 3, Student’s *t*-test); **G** lactate production (*n* = 4, Student’s *t*-test); **H** ADP/ATP ratio (*n* = 4, Student’s *t*-test); *p < 0.05, **p < 0.01, ***p < 0.001.

To validate our bioinformatics analyses of clinical osteosarcoma specimens (Figs. 1 and 2), we cultured six osteosarcoma cell lines with varying tumor aggressiveness under tumorsphere conditions (for OSCs) and subsequently determined *PDK1* mRNA levels (Fig. 3A) [46, 47]. Among these, *PDK1* mRNA expression was highest in MG-63, followed by 143B, Hu09, HOS, SJSA1, and Saos-2, suggesting a weak correlation between tumor aggressiveness and *PDK1* expression in OSCs (Fig. 3A). For further analysis, we selected the two cell lines with the highest *PDK1* expression: 143B and MG-63 (high and low—metastatic, respectively) [46]. First, we cultured 143B cells under tumorsphere conditions (143B OSCs) and adherent conditions (143B non-OSCs), followed by RNA-seq analysis. We previously demonstrated that 143B OSCs represent stemness properties and tumorigenicity in vitro and in vivo [34]. We identified DEGs related to the early stage of energy metabolic pathways in 143B OSCs (Fig. 3B). The expression of *PDK1* was significantly upregulated in 143B OSCs rather than in 143B non-OSCs (Fig. 3C), consistent with findings in the OSC population of primary osteosarcoma specimens (Fig. 2B), along with significant upregulation of stem cell markers, such as *SOX2*, *KLF4*, and *ABCG1* (Fig. 3D) [48–52]. Moreover, the protein level of PDK1 was significantly upregulated in both 143B and MG-63 OSCs, concomitant with higher protein levels of stem cell markers SOX2 and c-MYC (Fig. 3E) [48, 49, 53]. Additionally, glucose uptake and lactate production were significantly increased in 143B OSCs (Fig. 3F, G), while the ADP/ATP ratio was significantly decreased (Fig. 3H), indicating glycolysis activation in OSCs over differentiated osteosarcoma cells. These findings suggest that OSCs may favor glycolysis through PDK1 overexpression, highlighting PDK1’s role in regulating OSC stem cell phenotypes and tumorigenicity.

### Genetic inhibition of *PDK1* reduces OSC stemness and glycolysis in vitro

**Fig. 4:**
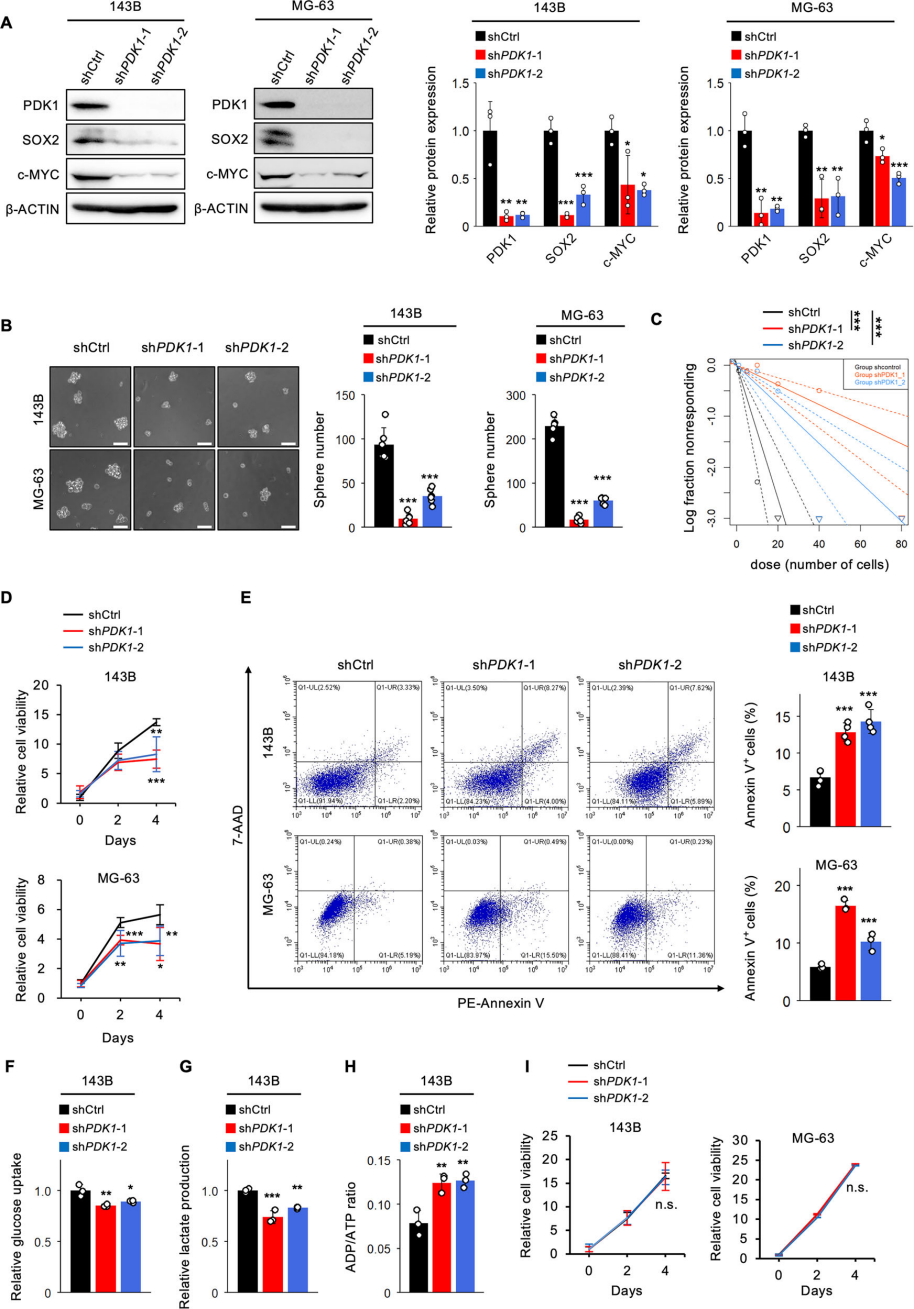
*PDK1* knockdown diminishes OSCs stemness and glycolysis in vitro. **A**–**E** Analysis of *PDK1* knockdown in OSCs derived from 143B and MG-63 cells; **A** western blot of PDK1 and stem cell markers (*n* = 3–6, one-way ANOVA followed by Dunnett’s test); **B** sphere formation assay (*n* = 6, one-way ANOVA followed by Dunnett’s test, scale bar: 30 μm); **C** in vitro limiting dilution assay (*n* = 10); **D** MTT assay for cell proliferation (*n* = 5, two-way ANOVA with Tukey–Kramer test); **E** apoptosis assay with PE-Annexin V and 7-AAD staining (*n* = 3–4, one-way ANOVA followed by Dunnett’s test); **F**–**H** analysis of glycolytic activity; **F** glucose uptake (*n* = 3, one-way ANOVA followed by Dunnett’s test); **G** lactate production (*n* = 4, one-way ANOVA followed by Dunnett’s test); **H** ADP/ATP ratio (*n* = 4, one-way ANOVA followed by Dunnett’s test); **I** MTT assay for cell proliferation in non-OSCs (*n* = 5, two-way ANOVA with Tukey–Kramer test); *p < 0.05, **p < 0.01, ***p < 0.001.

We targeted *PDK1* in 143B and MG-63 OSCs using lentiviral shRNAs (sh*PDK1*#1 and sh*PDK1*#2). Knockdown of *PDK1* significantly reduced SOX2 and c-MYC protein levels in both cell lines, along with PDK1 itself (Fig. 4A). Sphere formation assays showed that *PDK1* silencing markedly decreased tumorsphere formation in both 143B and MG-63 OSCs (Fig. 4B). An in vitro limiting dilution assay revealed that *PDK1* silencing significantly impaired OSC self-renewal (Fig. 4C). *PDK1* targeting reduced cell proliferation (Fig. 4D) and increased apoptosis in both (Fig. 4E). Additionally, *PDK1* disruption significantly decreased migration potential (Supplementary Fig. 3A). *PDK1* knockdown also reduced glucose uptake and lactate production while increasing the ADP/ATP ratio in 143B OSCs, indicating reduced glycolysis (Fig. 4F–H). Contrary to its effects under tumoresphere conditions, *PDK1* silencing did not considerably affect cell proliferation or apoptosis in 143B and MG-63 cells under adherent conditions (Fig. 4I and Supplementary Fig. 3B). These findings suggest that PDK1 is crucial for maintaining OSC stem cell properties and metabolic preferences in vitro.

### Targeting *PDK1* reduces OSC tumorigenicity and stemness in vivo

**Fig. 5:**
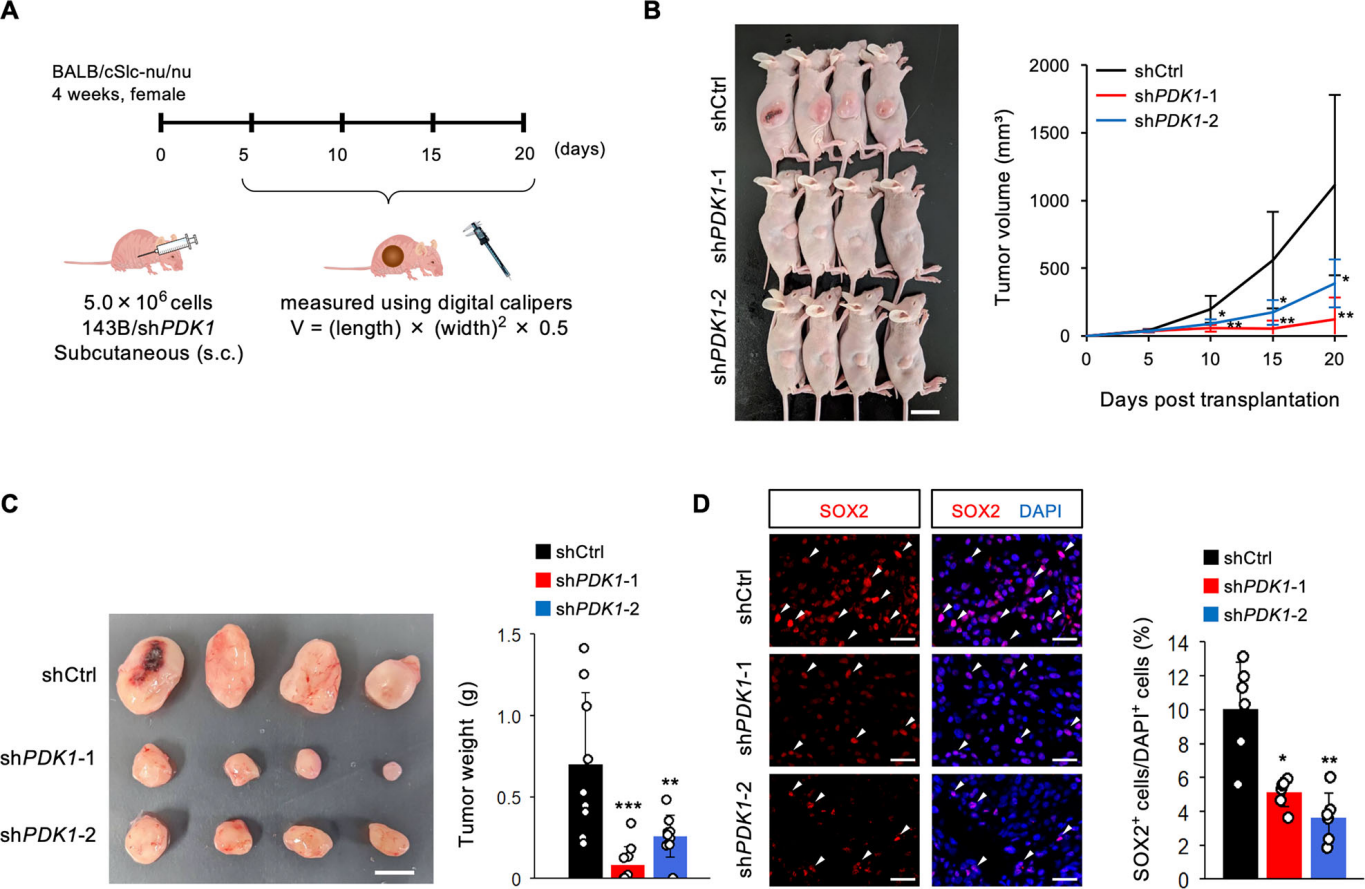
*PDK1* knockdown reduces tumorigenicity and stemness in vivo. **A**–**D** in vivo analysis using a xenograft model; **A** schematic showing subcutaneous transplantation of *PDK1* knockdown 143B cells (5 × 10^6^ cells/mouse) into 4-week-old female BALB/cSlc-nu/nu mice; **B** tumor volume measurement (*n* = 9–10, two-way ANOVA with Tukey–Kramer test, scale bar: 20 mm); **C** tumor weight (*n* = 9–10, one-way ANOVA followed by Dunnett’s test, scale bar: 10 mm); **D** immunohistochemical analysis of SOX2 expression in osteosarcoma tissues (*n* = 4, one-way ANOVA followed by Dunnett’s test, scale bar: 30 μm); *p < 0.05, **p < 0.01, ***p < 0.001.

Given PDK1’s role in OSC growth, survival, aggressiveness, and self-renewal in vitro, we investigated its effect on tumorigenicity in a heterotopic xenograft mouse model (Fig. 5A). Equal numbers of 143B OSCs infected with sh*PDK1* or shControl (shCtrl) were subcutaneously transplanted into immunocompromised mice. Mice with sh*PDK1*-infected 143B OSCs had significantly lower tumor volume and weight compared to those with shCtrl-infected OSCs (Fig. 5B, C). Histological analysis showed a significant reduction in SOX2-positive cells in tumors from sh*PDK1*-infected OSCs compared to shCtrl-infected OSCs (Fig. 5D). These results indicate that PDK1 is essential for maintaining tumorigenicity and stemness in vivo.

### Pharmacological inhibition of PDK1 suppresses OSC stemness, tumorigenicity, and metastasis in vitro and in vivo

**Fig. 6:**
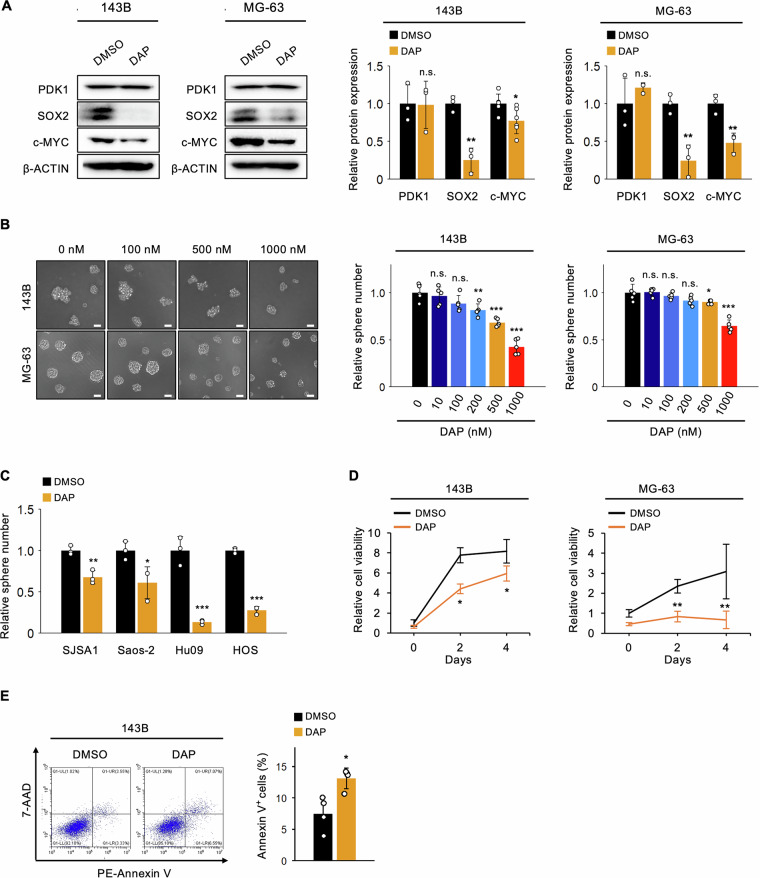
Pharmacological inhibition of PDK1 reduces OSC stemness in vitro. Analysis of DAP-treated OSCs derived from 143B and MG-63 cells; **A** western blot showing SOX2 and c-MYC protein levels (1 μM DAP, *n* = 3, Student’s *t*-test); **B** sphere formation assay (*n* = 6, Student’s *t*-test, scale bar: 30 μm); **C** sphere formation assay in OSCs of SJSA1, Saos-2, Hu09 and HOS cells (1 μM DAP, *n* = 3, Student’s *t*-test); **D** MTT assay for cell proliferation (1 μM DAP, *n* = 5, two-way ANOVA with Tukey–Kramer test); **E** apoptosis assay with PE-Annexin V and 7-AAD staining (1 μM DAP, *n* = 3, Student’s *t*-test); *p < 0.05, **p < 0.01, ***p < 0.001.

Our studies indicate that PDK1 in OSCs controls their stemness, tumorigenicity, and energy metabolism, making it a promising osteosarcoma therapy target. We tested whether DAP, a PDK1 inhibitor, could suppress OSC stemness and tumorigenicity [54]. DAP treatment significantly decreased SOX2 and c-MYC protein levels in 143B and MG63 OSCs without altering PDK1 levels (Fig. 6A). DAP also reduced tumor sphere formation dose-dependently in 143B and MG63 OSCs (Fig. 6B). DAP also significantly decreased tumor sphere formation in SJSA1, Saos-2, Hu09, and HOS under tumorsphere condition (Fig. 6C). Additionally, DAP decreased cell proliferation and increased apoptosis in OSCs (Fig. 6D, E).

**Fig. 7:**
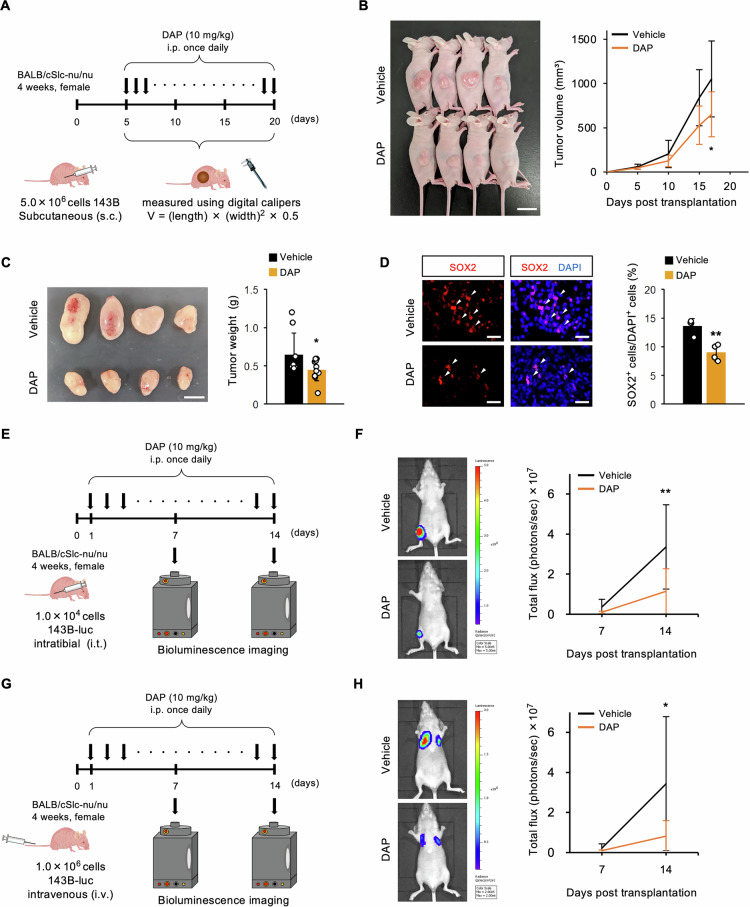
Pharmacological inhibition of PDK1 suppresses OSC stemness, tumorigenicity, and metastasis in vivo. **A**–**D** in vivo analysis in heterotopic xenograft mouse model; **A** schematic of subcutaneous transplantation of 143B cells (5 × 10^6^ cells/mouse) and DAP administration in 4-week-old female BALB/cSlc-nu/nu mice; **B** tumor volume (*n* = 10, two-way ANOVA with Tukey–Kramer test, scale bar: 20 mm); **C** tumor weight (*n* = 10, Student’s *t*-test, scale bar: 10 mm); **D** immunohistochemical analysis of SOX2 in tumor tissues (*n* = 4, Student’s *t*-test, scale bar: 30 μm); **E** and **F** in vivo analysis in orthotopic xenograft mouse model; **E** schematic of intratibial injection of 143B-luc cells (1 × 10^4^ cells/mouse) and DAP administration in 4-week-old female BALB/cSlc-nu/nu mice, followed by bioluminescent imaging; **F** tumor accumulation in tibia (*n* = 10, two-way ANOVA with Tukey–Kramer test); **G** and **H** in vivo analysis in pulmonary metastasis mouse model; **G** schematic of intravenous injection of 143B-luc cells (1 × 10^6^ cells/mouse) and DAP administration in 4-week-old female BALB/cSlc-nu/nu mice, followed by bioluminescent imaging; **H** tumor accumulation in lung (*n* = 10, two-way ANOVA with Tukey–Kramer test); *p < 0.05, **p < 0.01.

In a heterotopic xenograft mouse model, DAP was intraperitoneally administered to immunocompromised mice, which significantly reduced tumor volume and weight and SOX2-positive cells in tumors (Fig. 7A–D), indicating the reduction of OSC stemness and tumorigenicity. To investigate the microenvironmental dependence of PDK1-driven OSC phenotypes, we established an orthotopic xenograft model via intratibial injection of luciferase-expressing 143B OSCs (143B-luc) and a pulmonary metastasis model via intravenous injection of 143B-luc cells. Tumor burden and treatment response were evaluated using bioluminescent imaging. Daily administration of DAP notably reduced tumor accumulation in the tibia in the orthotopic xenograft model (Fig. 7E, F) and suppressed pulmonary metastasis development in the experimental metastasis model (Fig. 7G, H). Altogether, these results indicate that pharmacological inhibition of PDK1 effectively diminishes OSC stemness, tumorigenicity, and metastatic potential.

### PDK1-dependent metabolic adaptation governs OSC properties through ATF3

**Fig. 8:**
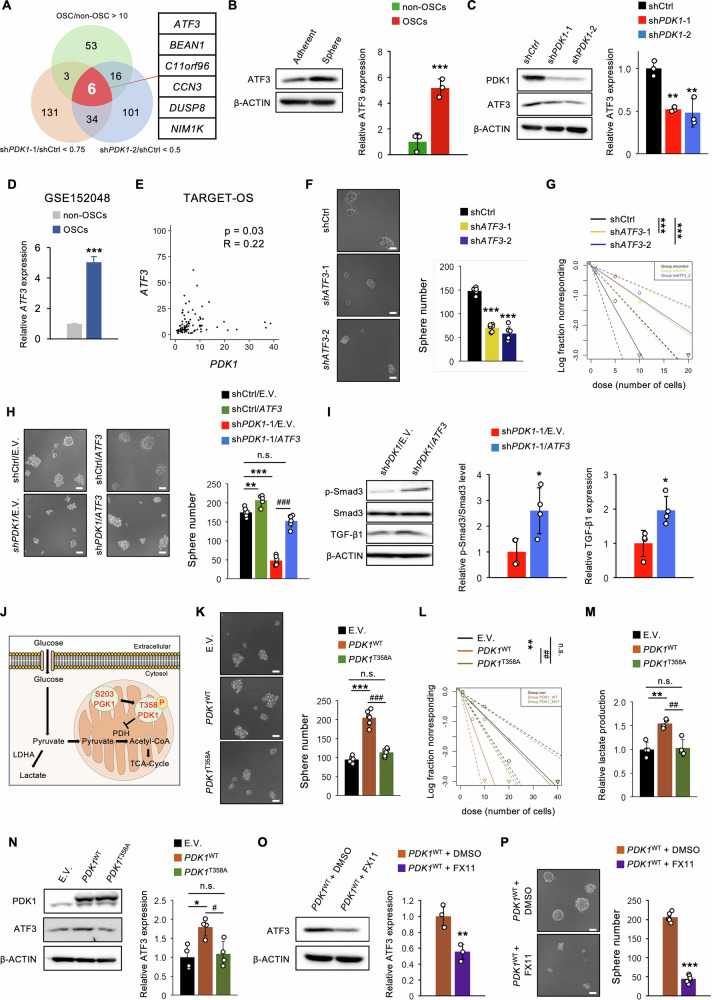
PDK1-dependent metabolic adaptation regulates OSC traits via ATF3. **A**–**I** and **K**–**P** Analysis of OSCs derived from 143B cells; **A** venn diagram displaying genes upregulated in OSCs and downregulated in *PDK1* knockdown OSCs from RNA-seq data; **B** western blot analysis comparing OSCs and non-OSCs (*n* = 3, Student’s *t*-test); **C** western blot analysis in *PDK1* knockdown OSCs (*n* = 3, one-way ANOVA followed by Dunnett’s test); **D** mRNA expression levels of ATF3 in OSCs (*n* = 250) and non-OSCs (*n* = 24,841); Wilcoxn test, mean ± standard error); **E** correlation analysis of *PDK1* and *ATF3* expression in the TARGET-OS cohort; **F** sphere formation assay in *ATF3* knockdown OSCs (*n* = 6, one-way ANOVA followed by Dunnett’s test); **G** in vitro limiting dilution assay for *ATF3* knockdown OSCs (*n* = 10); **H** sphere formation assay in sh*PDK1*/*ATF3* OSCs (*n* = 6, two-way ANOVA followed by Tukey–Kramer test); **I** western blot analysis in sh*PDK1*/*ATF3* OSCs (*n* = 4, Student’s *t*-test); **J** schematic showing glycolysis regulation by PGK1 and PDK1; **K** sphere formation assay in *PDK1*^*T358A*^ OSCs (*n* = 6, one-way ANOVA followed by Tukey–Kramer test); **L** in vitro limiting dilution assay for *PDK1*^*T358A*^ OSCs (*n* = 10); **M** lactate production measurement in *PDK1*^*T358A*^ OSCs (*n* = 4, one-way ANOVA followed by Tukey–Kramer test); **N** western blot analysis in *PDK1*^T358A^ OSCs. (*n* = 4, one-way ANOVA followed by Tukey–Kramer test); **O** western blot analysis in *PDK1*^WT^ and FX11-treated OSCs (20 μM FX11, *n* = 3, Student’s *t*-test); **P** sphere formation assay (20 μM FX11, *n* = 6, Student’s *t*-test); scale bar: 30 μm; *p < 0.05, **p < 0.01, ***p < 0.001, ^#^*p* < 0.05, ^##^*p* < 0.01, ^###^*p* < 0.001.

To explore PDK1’s control mechanisms, we identified DEGs in “*PDK1*-knockdown OSCs over control OSCs” and “OSCs over differentiated osteosarcoma cells” via RNA-seq. We found 174 and 157 downregulated genes in sh*PDK1*#1-infected 143B OSCs and sh*PDK1*#2-infected 143B OSCs, respectively, and 78 upregulated genes in OSCs (Fig. 8A). Six overlapping genes included *ATF3*, associated with stemness in glioma stem cells (GSCs) [55, 56]. ATF3 protein levels were significantly higher in 143B OSCs than in differentiated cells (Fig. 8B) and decreased in *PDK1*-knockdown OSCs (Fig. 8C). *ATF3* mRNA level was elevated in OSCs (GSE152048) (Fig. 8D), and TARGET-OS analysis showed a positive correlation between *PDK1* and *ATF3* expression in patients with osteosarcoma (Fig. 8E). *ATF3* silencing in 143B OSCs significantly reduced tumorsphere formation and self-renewal potential (Fig. 8F, G), along with the reduction of ATF3 protein levels (Supplementary Fig. 4A). Conversely, *ATF3* overexpression reversed the reduction in tumorsphere formation caused by *PDK1* knockdown (Fig. 8H and supplementary Fig. 4B). *ATF3* overexpression did not significantly alter PDK1 protein levels, lactate production, or ATP content in either control OSCs or *PDK1*-knockdown OSCs (Supplementary Fig. 4B–D). However, ATF3 has been reported to regulate GSC stemness via the TGF-β1 signaling pathway [55]. In *PDK1*-knockdown OSCs, *ATF3* overexpression upregulated TGF-β1 protein levels and Smad3 phosphorylation (Fig. 8I). Therefore, ATF3 partially compensates for the loss of stemness in *PDK1*-deficient OSCs by activating the TGF-β/Smad pathway, independent of metabolic alterations.

PDK1 is activated by phosphorylation at Thr358 by PGK1, leading to PDH inactivation, which converts pyruvate to acetyl-CoA (Fig. 8J) [25]. Overexpression of wild-type-*PDK1* (*PDK1*^WT^) significantly increased tumorsphere formation, self-renewal potential, and lactate production in 143B OSCs (Fig. 8K–M). Conversely, introducing kinase-dead (KD)-*PDK1* (*PDK1*^T358A^) did not significantly alter these properties (Fig. 8K–M, Supplementary Fig. 4E). OSC phenotypes were significantly reduced with *PDK1*^T358A^ compared to *PDK1*^WT^ (Fig. 8K, L). Further, ATF3 expression was significantly increased by *PDK1*^WT^ but not by *PDK1*^T358A^ in 143B OSCs, despite elevated PDK1 levels in both OSCs (Fig. 8N). Pharmacological inhibition of lactate dehydrogenase A by FX11 reversed ATF3 upregulation and tumorsphere formation in *PDK1*^WT^-overexpressing 143B OSCs (Fig. 8O, P). These results suggest that PDK1-mediated metabolic reprogramming governs OSC phenotypes partly through ATF3.

## Discussion

ATF3, a member of the ATF/CREB family of basic-leucine zipper transcription factors, modulates various cellular functions, including proliferation, apoptosis, and glucose metabolism [57]. It acts as both an oncogene and tumor suppressor, depending on the tumor type and context [55–60]. In HSCs, ATF3 is activated by tumor-primed bone marrow MSCs, redirecting hematopoiesis toward monocytic cell expansion [61]. In GSCs, ATF3 enhances the stemness and tumorigenicity via TGF-β/Smad signaling and promotes resistance to temozolomide by inducing ABCB4, an ABC transporter [55, 56]. While ATF3’s role in ferroptosis in osteosarcoma has been noted, its impact on the stemness and tumorigenicity of OSCs and osteosarcoma pathophysiology remains unexplored [62]. Herein, we identified ATF3 as a candidate factor involved in PDK1-regulated OSC phenotypes. ATF3 was shown to rescue the loss of OSC stemness due to *PDK1* deficiency, at least partially, by activating the TGF-β/Smad signaling pathway, without affecting the metabolic balance in OSCs. Moreover, we identified other candidate genes (*BEAN1*, *C11orf96*, *CCN3*, *DUSP8*, and *NIM1K*) that may contribute to the PDK1-dependent maintenance of OSC stemness. While further investigation is warranted, our findings position ATF3 as a key downstream effector in the PDK1-mediated regulation of OSC characteristics and osteosarcoma pathogenesis.

OSCs significantly contribute to metastasis and recurrence, which are major causes of poor outcomes in patients with osteosarcoma [4–6]. Our scRNA-seq analysis of clinical osteosarcoma specimens revealed that *PDK1* expression was significantly elevated in OSCs from primary tumors, but not in those from metastatic or recurrent samples. Herein, we utilized six osteosarcoma cell lines with differing characteristics: 143B, SJSA1, Saos-2, and Hu09 are highly-metastatic, whereas MG63 and HOS are lowly-metastatic [46]. OSC aggressiveness did not correlate well with either *PDK1* mRNA levels or its functional impact on stemness, as assessed across these cell lines. Nevertheless, pharmacological inhibition of PDK1 significantly reduced lung tumor burden in an experimental metastasis model, suggesting that PDK1 expression in OSCs may contribute to metastasis and the poor prognosis associated with osteosarcoma.

The metabolic characteristics of CSCs are highly heterogeneous. Unlike non-CSCs, which mainly utilize glycolysis, CSCs rely on either glycolysis or OXPHOS in a context-dependent manner, with contradictory results reported for the same tumor entity [12, 14, 17, 18]. For example, GSCs rely on glycolysis for energy production and survival through increased glucose consumption by upregulating GLUT3, while also depending on OXPHOS via IMP2, an RNA-binding protein [20, 21]. This discrepancy is attributed to CSC heterogeneity, tumor microenvironment, and experimental strategies [63, 64]. Although we identified the PDK1–glycolysis–ATF3 axis as a key regulator of OSC properties and osteosarcoma malignancy, the precise mechanisms by which PDK1-glycolysis regulates ATF3 expression in OSCs remain unclear. We propose that PDK1-driven metabolic reprogramming may indirectly influence ATF3 expression, further promoting OSC stemness through the activation of the TGFβ-Smad signaling pathway. However, to reinforce the conclusions of this study, further research is needed to elucidate how a metabolic kinase like PDK1 modulates ATF3 expression in OSCs.

Extensive studies have explored direct glycolysis inhibition as a potential cancer treatment, but success rates have been low. For instance, hexokinase 2 inhibition with lonidamine did not significantly improve overall survival in several cancers, including breast and lung, and caused elevated toxicity [65, 66]. Glucose transport inhibitors like silibinin (silybin) also caused critical side effects without significant response in prostate cancer [67]. In contrast, our study demonstrated that pharmacological inhibition of PDK1 significantly reduced lung tumor burden in an experimental metastasis model and suppressed tumor growth in orthotopic and heterotopic xenograft mouse models in vivo. This was accompanied by repressing OSC stemness in vitro, thus supporting the potential applicability of PDK1 inhibition in osteosarcoma treatment. This is the first preclinical study to reveal the link between metabolic reprogramming in OSCs, their stemness and tumorigenesis, and osteosarcoma malignancy. Despite extensive research efforts, effective treatments for osteosarcoma have not advanced significantly in the past 4 decades [1–3]. Our findings enhance the understanding of osteosarcoma pathogenesis and OSC properties. They suggest that targeting energy metabolic reprogramming and the associated genes in OSCs could be a novel and effective approach for developing new treatments for osteosarcoma in humans.

## Supplementary information

Supplemental Figure

Original western blot data

## Data Availability

The GSE21257, GSE126209, GSE152048, GSE179681, and GSE297211 datasets are deposited in the Gene Expression Omnibus (GEO) database (https://www.ncbi.nlm.nih.gov/geo/). The PRJNA539828 dataset is deposited in the National Center for Biotechnology Information (NCBI) database (https://www.ncbi.nlm.nih.gov/). The TARGET-OS and TCGA-SARC datasets are deposited in the Genomic Data Commons data portal (https://portal.gdc.cancer.gov/).
