# Supplementary material for: PDK1-dependent metabolic reprogramming regulates stemness and tumorigenicity of osteosarcoma stem cells through ATF3
Source: Cell Death Dis. 2025 Jul 29;16(1):574. doi: 10.1038/s41419-025-07903-7 (PMC12307947; doi:10.1038/s41419-025-07903-7)
Supplement: Supplementary file 1 — Supplemental Figure [file 41419_2025_7903_MOESM1_ESM.pdf]

Supplemental Fig. 1

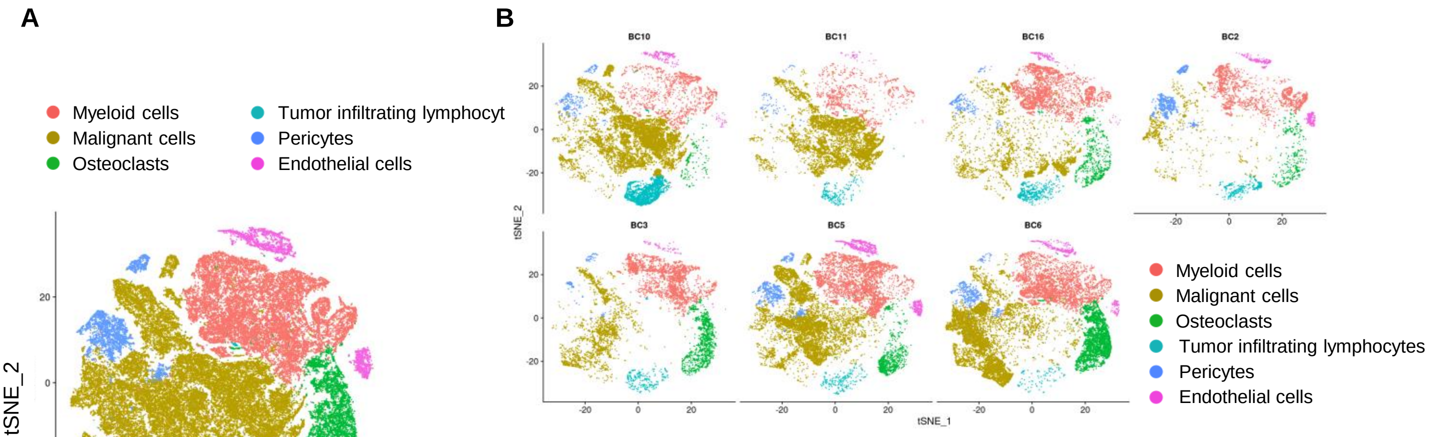

Sample	Pathological type	Type	Location
BC2	Conventional	Primary	Femur
BC3	Conventional	Primary	Tibia
BC5	Conventional	Primary	Femur
BC6	Conventional	Primary	Ulna
BC10	Conventional	Metastasis (Lung)	Femur
BC11	Conventional	Recurrent	Femur
BC16	Conventional	Primary	Tibia

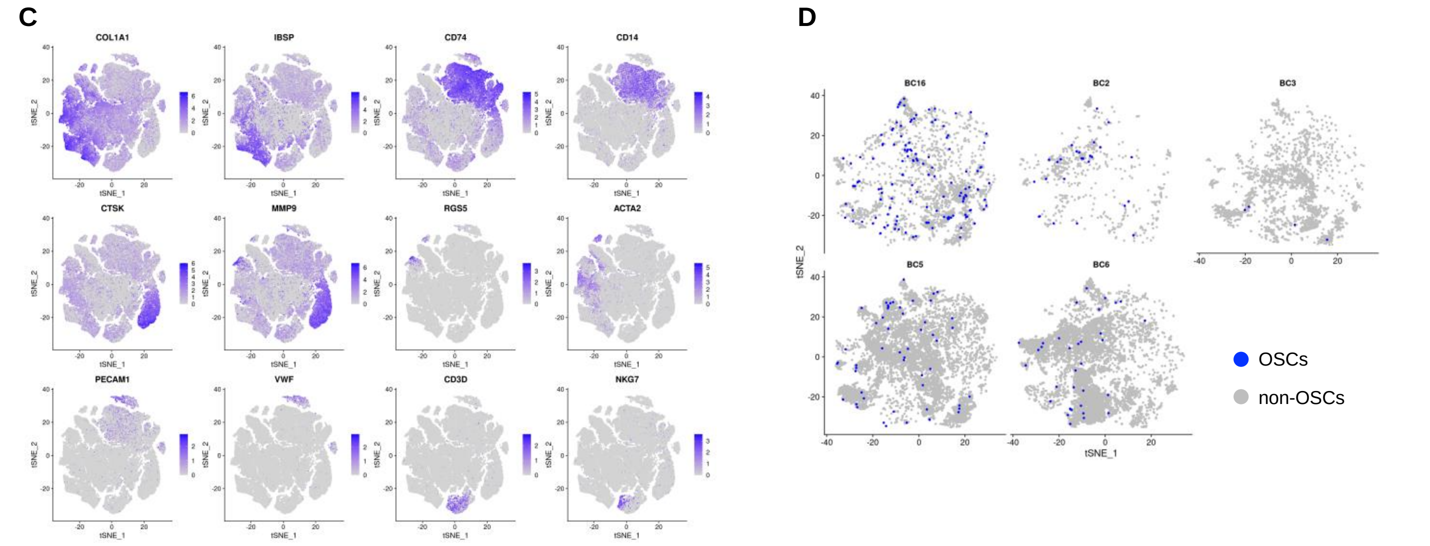

Supplemental Fig. 1

A t-distributed stochastic neighbor embedding (t-SNE) plot depicting six distinct cell clusters; **B** t-SNE plot showing cell clusters in each OS sample; **C** feature plots displaying canonical marker genes for identifying six cell clusters: malignant cells (*COL1A1*, *IBSP*), myeloid cells (*CD74*, *CD14*), osteoclasts (*CTSK*, *MMP9*), pericytes (*RGS5*, *ACTA2*), endothelial cells (*PECAM1*, *VWF*), and tumor-infiltrating lymphocytes (*CD3D*, *NKG7*); **D** t-SNE plot distinguishing OSCs and non-OSCs among malignant cells classified by ssGSEA in each primary sample

Supplemental Fig. 2

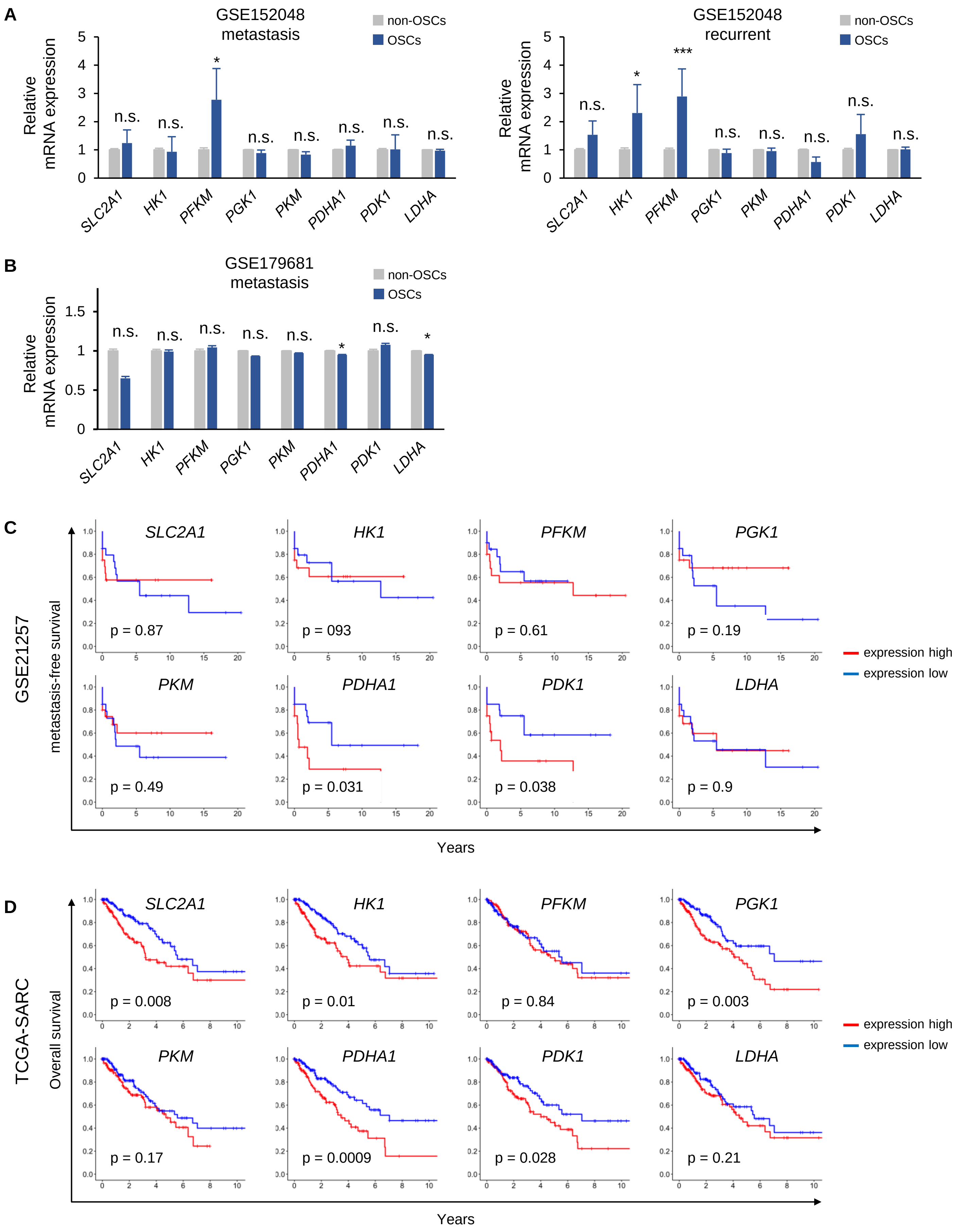

Supplemental Fig. 2

A and **B** mRNA expression levels of genes related to early energy metabolism from the **A** GSE152048 dataset; **B** GSE179681 dataset (Wilcoxon test, mean ± standard error); **C** and **D** The Kaplan–Meier survival curves showing survival rates for patients with osteosarcoma with high and low expression of early energy metabolic pathway genes; **C** metastasis-free survival in the GSE21257 cohort (high; n = 20, low; n = 20, Log-rank test); **D** overall survival in the TCGA-SARC cohort (high; n = 129, low; n = 130, Log-rank test)

Supplemental Fig. 3

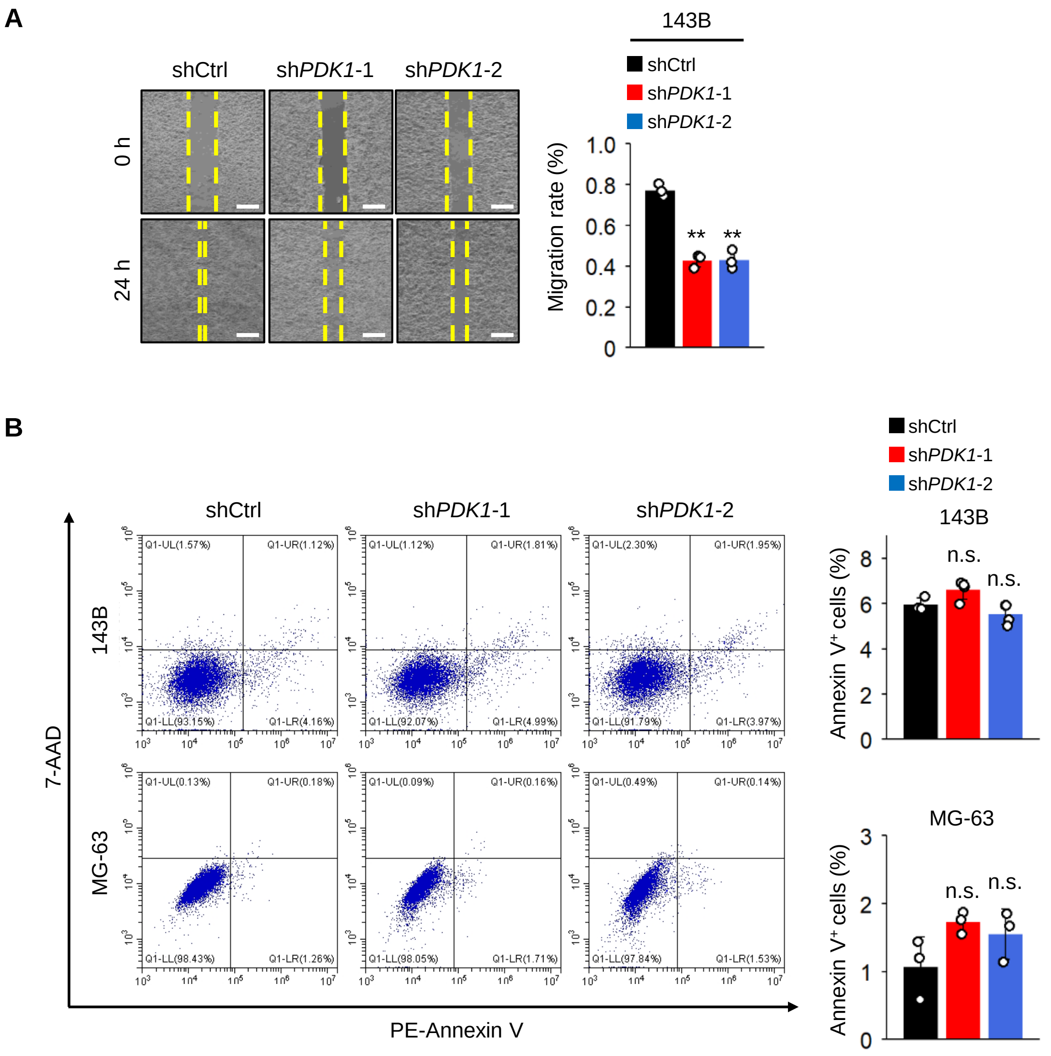

Supplemental Fig. 3

A wound healing assay for migration analysis in 143B OSCs (n = 3, one-way ANOVA followed by Dunnett's test, scale bar: 200 μm); **B** apoptosis assay in 143B and MG-63 non-OSCs with PE-Annexin V and 7-AAD staining (n = 3-4, one-way ANOVA followed by Dunnett's test); $**p < 0.01$

Supplemental Fig. 4

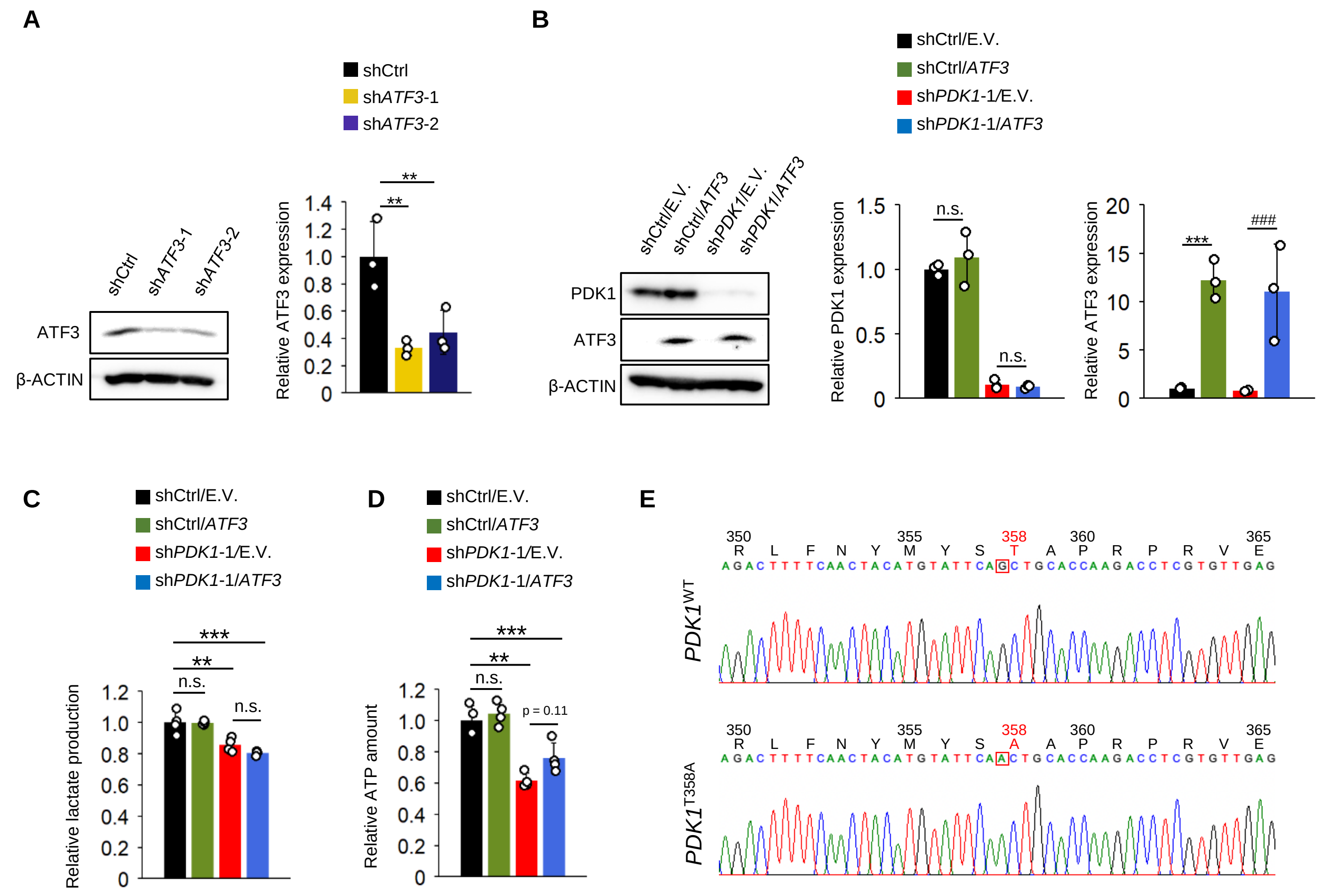

Supplemental Fig. 4

A western blot analysis in *ATF3* knockdown OSCs (n = 3, one-way ANOVA followed by Dunnett's test); **B** western blot analysis in sh*PDK1*/*ATF3* OSCs (n = 3, two-way ANOVA followed by Tukey–Kramer test); **C** lactate production in sh*PDK1*/*ATF3* OSCs (n = 4, two-way ANOVA followed by Tukey–Kramer test); **D** ATP amount in sh*PDK1*/*ATF3* OSCs (n = 4, two-way ANOVA followed by Tukey–Kramer test); **E** Nucleotide sequences of $PDK1^{WT}$ and $PDK1^{T358A}$; $^{**}p < 0.01$, $^{***}p < 0.001$, $^{\#\#\#}p < 0.001$
