# Supplementary figures and images for: PDK1-dependent metabolic reprogramming regulates stemness and tumorigenicity of osteosarcoma stem cells through ATF3
Source: Cell Death Dis. 2025 Jul 29;16(1):574. doi: 10.1038/s41419-025-07903-7 (PMC12307947; doi:10.1038/s41419-025-07903-7)

## E

## E

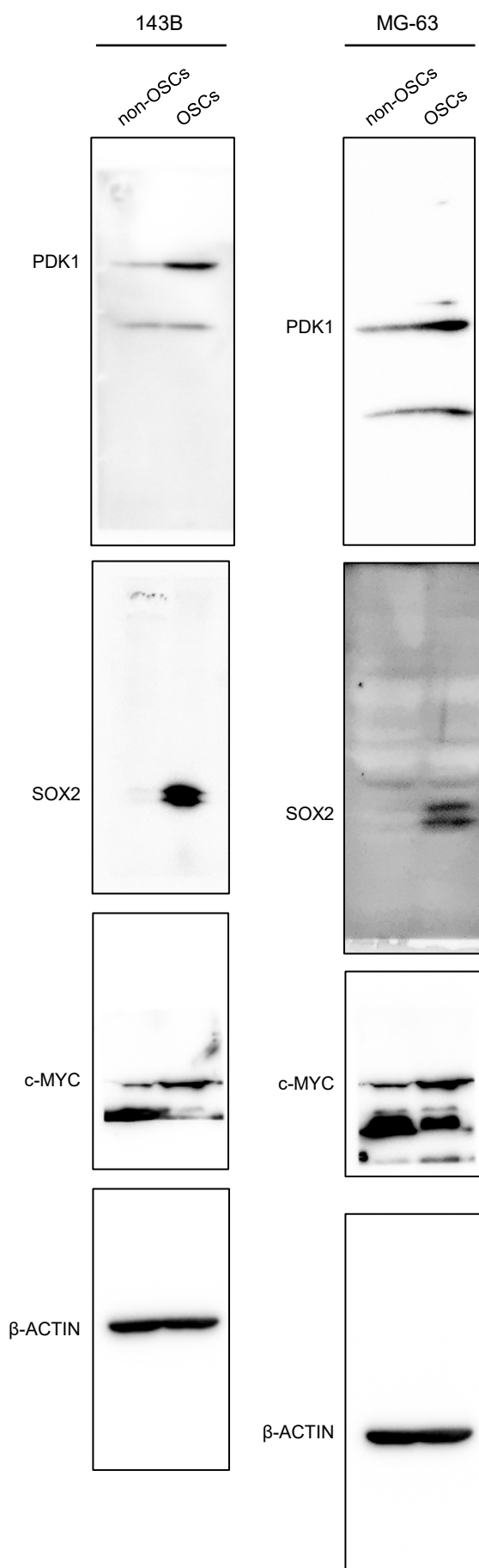

### Figure 4

**A**

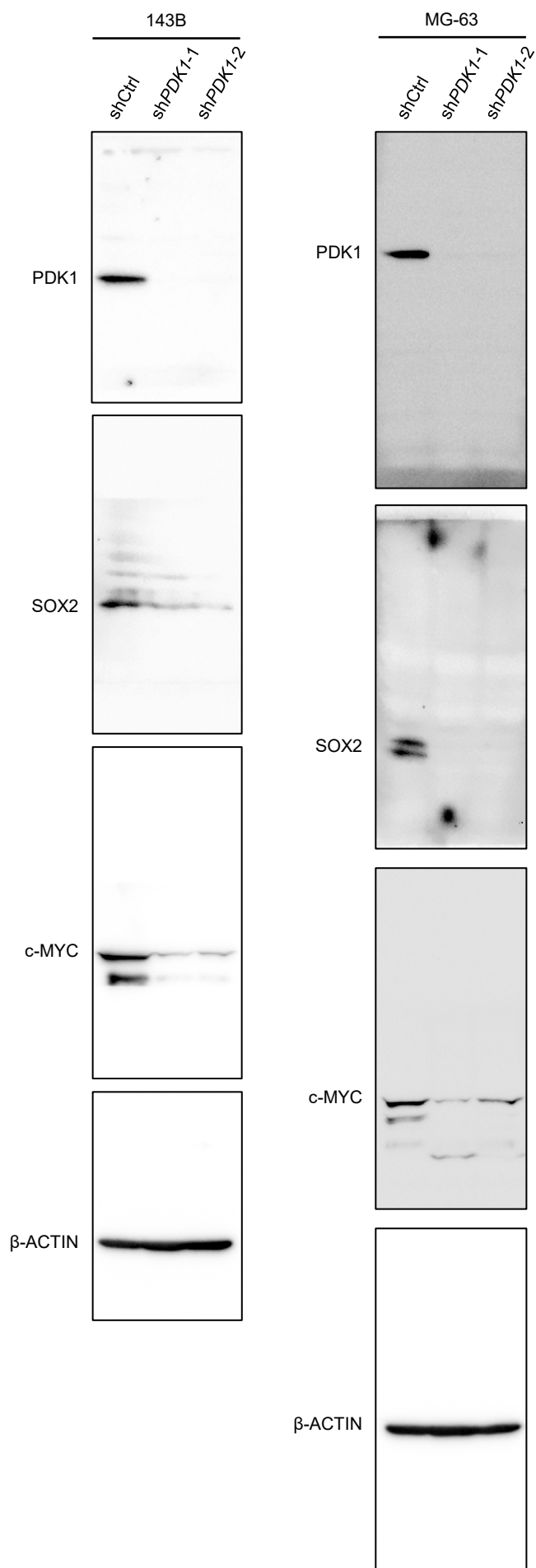

**Figure 6**

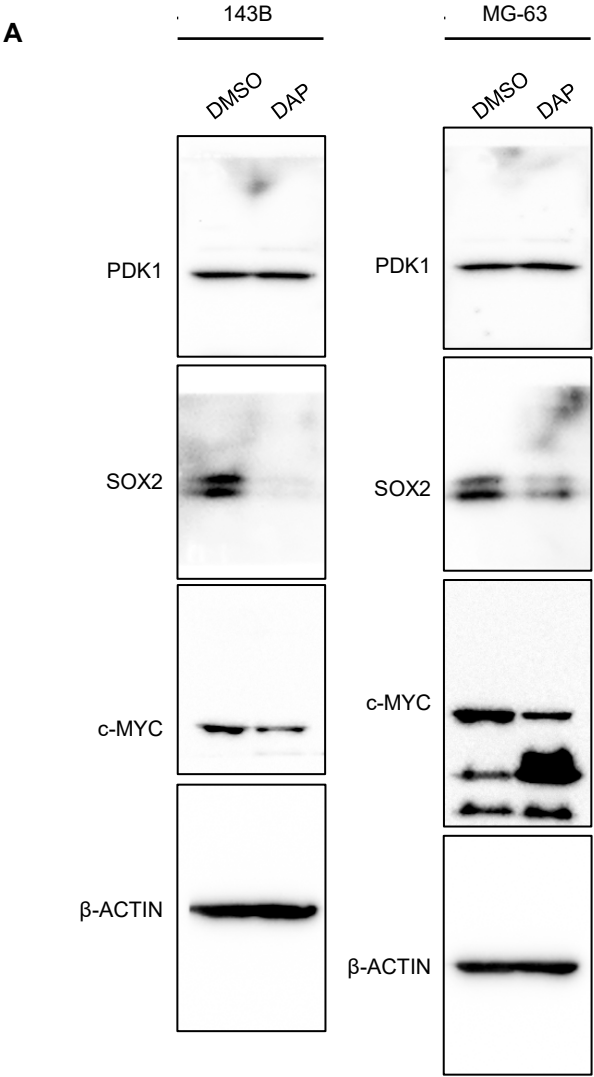

**Figure 8**

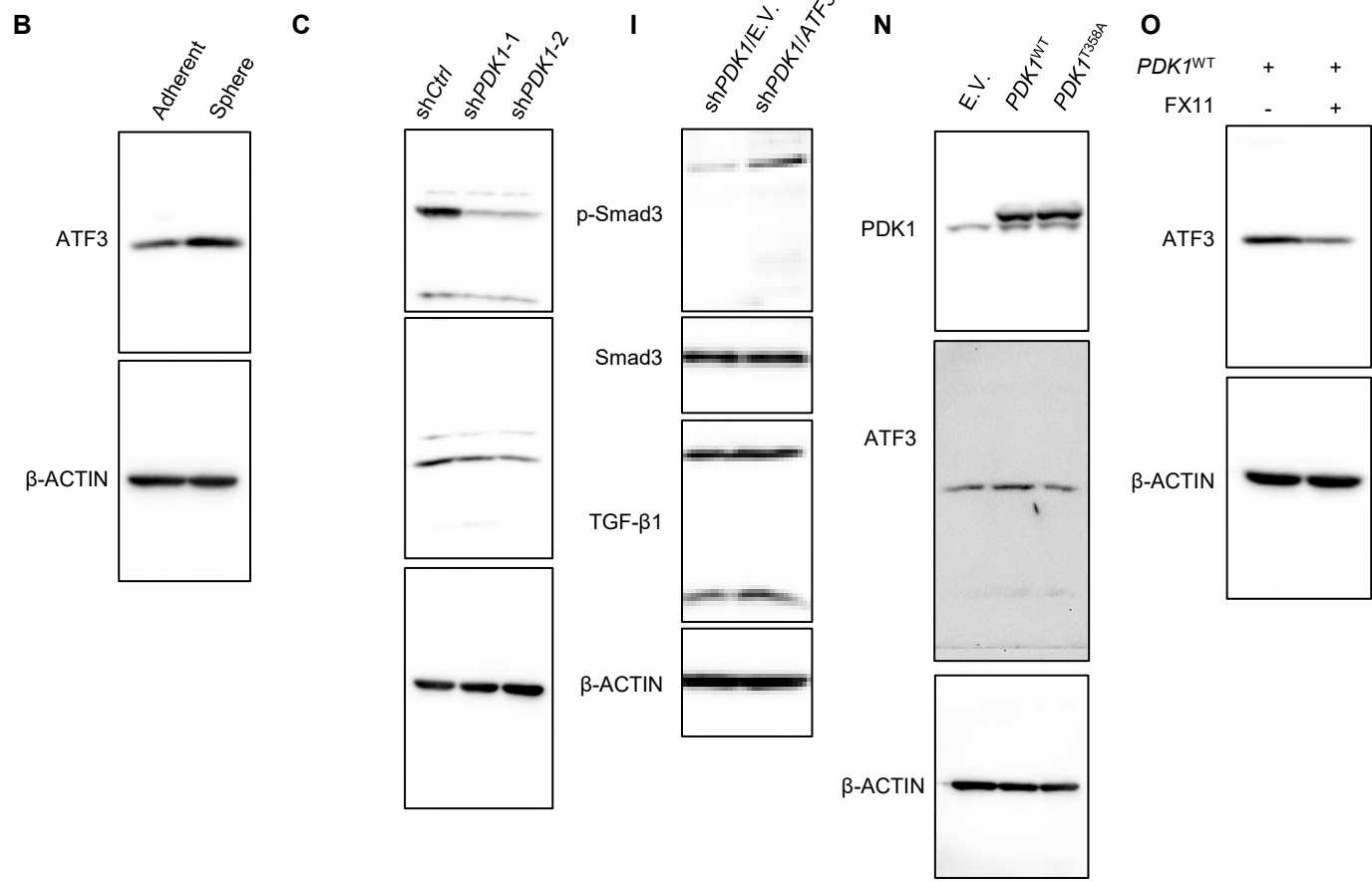

Supplemental Fig. 4

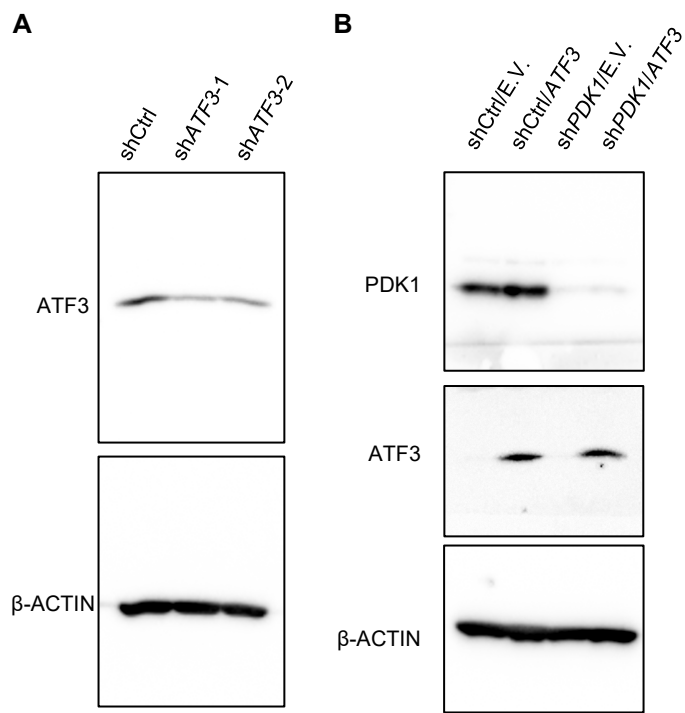

Supplement: Supplementary file 2 — Original western blot data [file 41419_2025_7903_MOESM2_ESM.pdf]
